# Cellular and Molecular Mechanisms of Spinal Cord Vascularization

**DOI:** 10.3389/fphys.2020.599897

**Published:** 2020-12-21

**Authors:** Jose Ricardo Vieira, Bhavin Shah, Carmen Ruiz de Almodovar

**Affiliations:** ^1^European Center for Angioscience, Medicine Faculty Mannheim, Heidelberg University, Mannheim, Germany; ^2^Faculty of Biosciences, Heidelberg University, Heidelberg, Germany; ^3^Interdisciplinary Center for Neurosciences, Heidelberg University, Heidelberg, Germany

**Keywords:** neurovascular, angiogenesis, spinal cord, VEGF, neural progenitors, blood brain barrier, CNS pathology

## Abstract

During embryonic central nervous system (CNS) development, the neural and the vascular systems communicate with each other in order to give rise to a fully functional and mature CNS. The initial avascular CNS becomes vascularized by blood vessel sprouting from different vascular plexus in a highly stereotypical and controlled manner. This process is similar across different regions of the CNS. In particular for the developing spinal cord (SC), blood vessel ingression occurs from a perineural vascular plexus during embryonic development. In this review, we provide an updated and comprehensive description of the cellular and molecular mechanisms behind this stereotypical and controlled patterning of blood vessels in the developing embryonic SC, identified using different animal models. We discuss how signals derived from neural progenitors and differentiated neurons guide the SC growing vasculature. Lastly, we provide a perspective of how the molecular mechanisms identified during development could be used to better understand pathological situations.

## Spinal Cord Development

The development of the central nervous system (CNS) of vertebrates starts with the formation of the neural tube, an ectoderm-derived embryonic structure that gives rise to the brain and the spinal cord (SC). In mouse, this process starts at E7.5–E8 (embryonic day) and, as the neural tube grows and matures along the anterio-posterior axis, it partitions into the rostral and caudal vesicles. While the rostral vesicles form the prosencephalon, mesencephalon, and the rhombencephalon (together, the brain), the caudal vesicle matures into the SC (Lumsden and Krumlauf, 1996; Purves, 2008). At around E9.5, the neural tube acquires a dorsal-ventral patterning by the controlled secretion of morphogens in a time and concentration dependent manner, establishing 13 different neural progenitor domains (Jessell, 2000; Dessaud et al., 2008). The identity of these progenitors is characterized by a unique expression profile of transcription factors, essential for the specification of the different neuronal subtypes that each domain generates. These individual expression profiles result from the combination of different morphogens: the floor plate and the notochord generate a gradient of Sonic Hedgehog (SHH), the key player for the ventral axis, while the roof plate creates a gradient of Wingless-related integration site (WNTs) family members and bone morphogenic proteins (BMPs), required for dorsal SC patterning. Other important factors, such as fibroblast growth factors (FGFs) and retinoic acid (RA), have been shown to contribute to a correct positioning of the neural progenitors (Diez del Corral et al., 2003; Duester, 2008; Diez Del Corral and Morales, 2017). For more details on neural development of the SC, we refer the readers to excellent reviews (Jessell, 2000; Wilson and Maden, 2005; Dessaud et al., 2007, 2008; Sagner and Briscoe, 2019).

Interestingly, as the neural progenitors undergo active proliferation and differentiation, the developing CNS starts to be simultaneously vascularized to meet the increasing demand of nutrients and oxygen. The avascular CNS starts to be vascularized at E8.5–E9.5 in mice by the formation of the perineural vascular plexus (PNVP) from mesoderm-derived angioblasts that completely covers the entire neural tube (Hogan et al., 2004; Engelhardt and Liebner, 2014). Between E9.5 and E10.5, the first blood vessels from the PNVP start ingressing into the developing SC in a stereotypical growth pattern (Nakao et al., 1988; Himmels et al., 2017). Unlike the SC, where vessels ingress from a single vascular plexus, the neocortex is vascularized by the ingression of vessels from the PNVP and an additional periventricular plexus (PVP) (Vasudevan et al., 2008). There, intrinsic transcriptional factors (Nkx2.1, Dlx1/2, and Pax6) expressed in endothelial cells are crucial for such vessel patterning from the PVP (Vasudevan et al., 2008).

Research from the last decade suggests that during development the nervous and vascular systems actively and vitally crosstalk with each other to build a fully functional organ system (Paredes et al., 2018; Segarra et al., 2019). In the next sections, we will focus on those interactions that occur during SC development in coordination with vascularization. From the current knowledge of developmental studies in SC using different animal models, we also introduce the readers with SC pathologies and the molecular targets discovered during development that could prove beneficial during injuries and diseases.

## Spinal Cord Vascularization: From a Simple Plexus to a Complex Network

### Development of the PNVP

The early development of the SC vasculature begins at around E8.5 in mouse embryos and around E3 in avian embryos with the formation of the PNVP, a primitive vascular network formed *via* the process of vasculogenesis (Noden, 1988; Kurz et al., 1996). The assembling of this vascular bed initially requires proliferation, migration, and differentiation of mesoderm-derived angioblasts, a mesenchymal cell type giving rise to the endothelial cell lineage (Noden, 1988; Kurz et al., 1996). The identification of the first key factors contributing for this process, FGF-2 and vascular endothelial growth factor (VEGF), was initially identified by experimentation in quail (Cox and Poole, 2000; Hogan et al., 2004). In avian and mouse embryos, the first primordial vascular structures neighboring the SC and fusing to the PNVP are the dorsal aorta (the major artery) and the cardinal vein (the major vein). Mice and avians present two dorsal aortas, one dorsal aorta in each side of the midline (defined by the notochord) – but fusing into a single artery in the mid-trunk region – and two cardinal veins, similarly one in each side of the midline along the entire body (Hogan and Bautch, 2004; Kurz, 2009). In avians, primitive arterial tracts connect each of the dorsal aortas to the ventral part of the PNVP, laterally to the FP, precisely where the first sprouts from the PNVP ingress into the SC. Afterward, the SC vascular circuit is closed and drained into the cardinal veins, which are connected to the PNVP in the lateral sides of the SC (Kurz, 2009). For the best of our knowledge, a similar vascular circuit in mouse has not been demonstrated yet.

In zebrafish, the PNVP forms, however, a bit different. In zebrafish, the dorsal aorta and the cardinal vein are single vessels located below the notochord and extend throughout the entire anterior-posterior axis. The PNVP surrounding the SC arises by the combination of sprouts from arterial and venous intersegmental vessels (ISVs; which sprout from the previously formed dorsal aorta and posterior cardinal vein) and vertebral arteries (VTAs; Isogai et al., 2001, 2003; Matsuoka et al., 2016, 2017; Wild et al., 2017). Bilateral VTAs are formed along the SC by sprouting from the previously established ISVs. Genetically-ablation studies have shown that this process in zebrafish is regulated by VEGF secreted from radial glia cells located in the SC (Matsuoka et al., 2017). Interestingly, not only radial glia but also SC neurons were also shown to similarly control sprouting of venous ISVs around the developing SC in zebrafish (Wild et al., 2017). SC neurons simultaneously secrete VEGF and SFLT1 and, in case of a shift in the balance toward the former, ectopic sprouting of venous ISVs (not arterial ISVs) arise and surround the SC, suggesting that the VEGF signaling affects specifically venous ECs during this development stage window (Wild et al., 2017).

### Vessel Ingression Into the SC

In avians, single angioblasts are able to invade and migrate into the SC and thus contribute, together with the sprouting from the PNVP, to SC vascularization (Kurz et al., 1996). In mice, in contrast, once the PNVP is formed, vessel ingression into of the SC only occurs from new sprouts arising from the PNVP – *via* sprouting angiogenesis (Nakao et al., 1988; Himmels et al., 2017).

**Figure 1:**
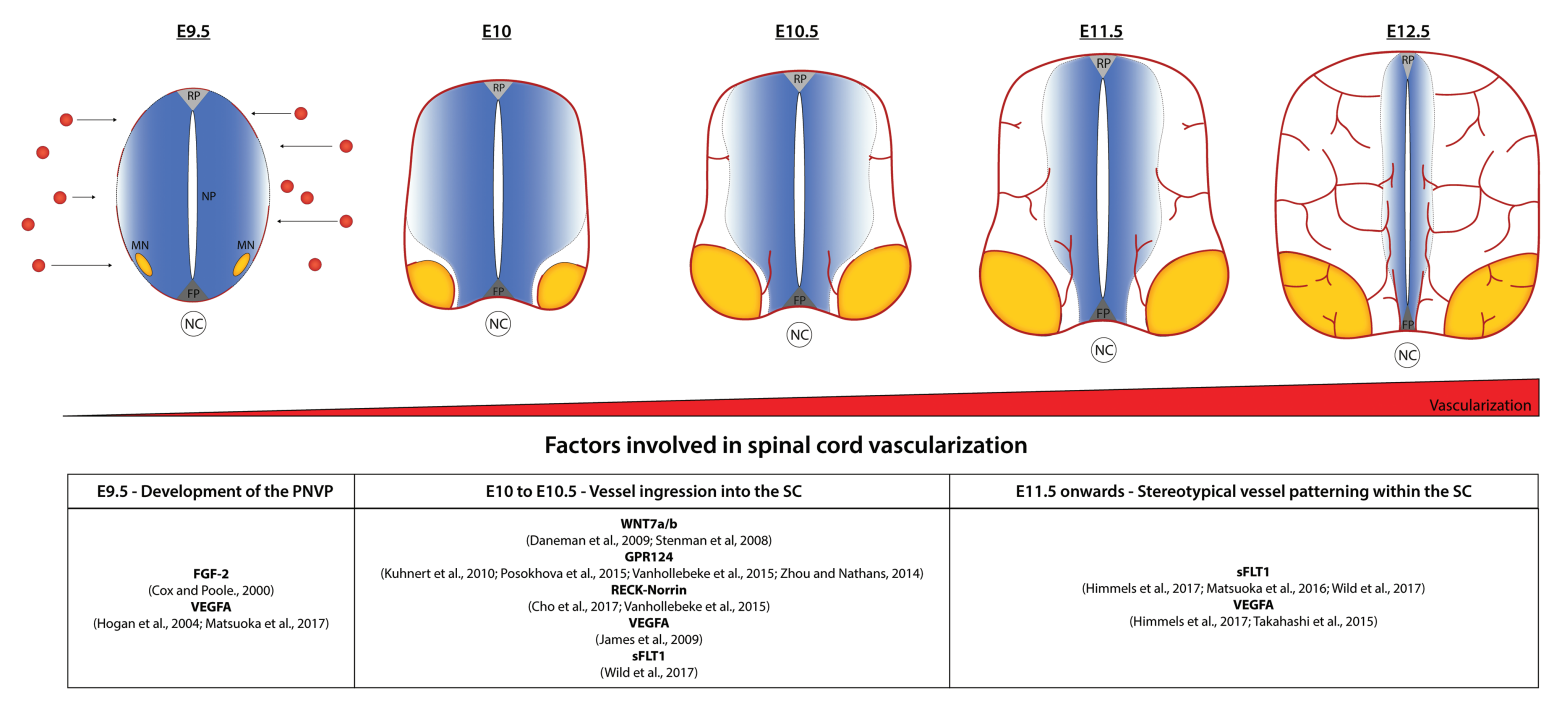
Stereotypical patterning of spinal cord (SC) vascularization. In normal conditions, the avascular SC starts to be vascularized at embryonic day 8.5–9.5 in mice by the formation of the perineural vascular plexus (PNVP). Between E9.5 and E10.5, blood vessels ingressing from the PNVP follow a specific invading pattern: the first vessel sprouts enter between the floor plate (FP) and motor neuron (MN) columns; subsequently they surround the MN columns and continue growing towards dorsal areas, but continuously avoiding the MNs region, the FP and part of the neural progenitors’ area (NP) for a particular development time window. At E12.5 MN columns are finally vascularized and a dense network of vessels sustains the continuous growth of the SC. Factors involved in the different steps are indicated in the table.

Blood vessel ingression into the SC occurs in a highly stereotypical way. At around E10.5 in mouse embryos, sprouting blood vessels from the PNVP invade the neural tissue in a very specific pattern: the first sprouts invade the ventral part of the SC in between the floor plate and MN columns and also from the lateral-ventral side in proximity with the MNs (Figure 1). VEGF, demonstrated to be important for PNVP formation, also plays a role in this initial blood vessel ingression. Interestingly, different VEGFA isoforms perturbed SC angiogenesis at different levels. While ectopic expression of VEGFA165 and VEGFA189 in quail embryos resulted in a considerable increase of vessel ingression, only a mild phenotype is observed after ectopic expression of VEGFA121 (James et al., 2009). Although VEGFA plays a role in blood vessel ingression, its expression is not specifically localized to areas of ingression, instead is broadly expressed in dorsal and ventral areas of the SC. This suggests that VEGFA is necessary but may not be the only factor controlling the location and timing of blood vessel sprouting from the PNVP (James et al., 2009). In fact, recent studies show that sFLT1 expression is also important to control the initial vascular sprouting into the SC in mouse (Himmels et al., 2017) and zebrafish (Wild et al., 2017). SC neurons (MNs in the case of mouse embryos) simultaneously secrete VEGF and sFLT1 to control the regions and timing of vessel ingression from the PNVP (Himmels et al., 2017; Wild et al., 2017).

WNT ligands play a major role in the initial sprouting of vessels into the SC. WNT7a and WNT7b, as other family members of the WNT family, are expressed in specific regions of the SC coincident with the time of ingression of the first vessel sprouts (Parr et al., 1993; Hollyday et al., 1995; Stenman et al., 2008; Daneman et al., 2009). WNT7a/b are expressed by several ventral and dorsal neural progenitors surrounding the ventricle at E10.5 and, while single null mutants for WNT7a or WNT7b do not present any phenotype, WNT7a/b double mutants show defects in their CNS-specific vasculature with the embryos dying around E12.5 (Stenman et al., 2008; Daneman et al., 2009). Remarkably, double mutant embryos were completely devoid of vessels and pericytes in the ventral part of the SC, but presented vessels (however with abnormal morphology) in dorsal regions of the SC. The fact that vessels continue to ingress into dorsal areas after deletion of WNT7a/b might suggested a role in SC vascularization for other WNT ligands expressed by the dorsal domains (Stenman et al., 2008). Consistently, blocking the canonical WNT signaling pathway by the removal of β-catenin from endothelial cells results in a more severe phenotype with the complete absence of blood vessels in the entire SC (Stenman et al., 2008; Daneman et al., 2009). Interestingly, in those mutants PNVP formation occurs normally, indicating that WNT signaling plays a role in the initial vessel ingression but not in PNVP formation (Stenman et al., 2008). Additionally, WNT7a/b also promote blood-brain barrier (BBB) formation, as lack of WNT7 leads to a reduction of GLUT1, the main glucose transporter in ECs of the CNS (Stenman et al., 2008). Wnt7a/b signal *via* Frizzled receptors (Zerlin et al., 2008) and RECK (reversion-inducing cysteine-rich protein), a GPI-anchored plasma membrane protein, was shown to act as a specific co-receptor for WNT7a/b in ECs (Vanhollebeke et al., 2015; Ulrich et al., 2016; Cho et al., 2017).

GPR124 is expressed by endothelial cells and pericytes and GPR124 null mice present severe hemorrhages as early as E12.5 and embryonic lethality at E15.5 (Kuhnert et al., 2010). Interestingly, GPR124 null mice present the same developmental defects observed in WNT7a/b and β-catenin null mice, suggesting that both signaling pathways share a common mechanism (Kuhnert et al., 2010). Indeed, independent groups showed that GPR124 acts as a co-activator of WNT7a/b-specific canonical pathway in endothelial cells (Zhou and Nathans, 2014; Posokhova et al., 2015; Vanhollebeke et al., 2015). Further studies have shown that a synergetic action of RECK-Norrin-GPR124 receptors in WNT7a/b signaling to promote vascular development, and the absence of either one is enough to reduce, but not completely abolish, WNT7a/b signaling (Vanhollebeke et al., 2015; Cho et al., 2017).

### Vessel Patterning Within the SC

As indicated above, the first vessel sprouts invade the ventral part of the SC in between the floor plate and MN columns and also from the lateral-ventral side in proximity with the MNs (Figure 1). At E11.5, vessels ingressing from both sites migrate toward each other and completely surround the MN columns, whereas at the same time additional sprouts branch from the previous vessels and also extend into dorsal areas of the SC, always along and in close contact with the ventricular zone occupied by the neural progenitors. Initially vessels do not ingress from the most dorsal part of the PNVP (Feeney and Watterson, 1946; James et al., 2009; Himmels et al., 2017). A considerable amount of research has been developed to try to understand this well-defined vascular patterning but still little is known about how the neural cells and growing blood vessels communicate to each other to orchestrate a correct SC vascularization pattern. Interestingly, we and others found that it is in the areas that blood vessels initially avoid (the floor plate, neural progenitors, and MN columns), where the highest amount of VEGF is detected (James et al., 2009; Ruiz de Almodovar et al., 2011; Himmels et al., 2017). This suggested that together with VEGF other factors were involved in blood vessel patterning. In line with this idea, we demonstrated how MNs control their own vascularization during a particular development time window (E10.5–E12.5) by simultaneously secreting VEGF and its decoy receptor sFLT1 in a hypoxia-inducible factor 1 (HIF1α) and neuropilin-1 (NRP1) dependent manner, respectively (Himmels et al., 2017). The importance of a perfect balance of these molecules is visible when overexpressing of VEGF in neural cells in mouse embryos or when reducing sFLT1 or NRP1 by knocking down their expression using *in ovo* experiments. Under those different conditions premature blood vessel ingression into MN columns occurs, showing an example of a neural-to-vessel communication mechanism that shapes SC development (Himmels et al., 2017). The importance of the balance VEGF-sFLT1 for a correct SC vascularization has also been described in zebrafish (Matsuoka et al., 2016, 2017; Wild et al., 2017).

As mentioned above blood vessels ingress from the ventral region and migrate toward dorsal areas along a well-defined path: in mice, vessels grow in close contact to the undifferentiated neural progenitors next to the ventricle while in avian vessels extend in between the undifferentiated neural progenitor zone (Sox2^+^) and the differentiated zone (Sox2^−^ Tuj-1^+^; Takahashi et al., 2015; Himmels et al., 2017). Manipulation of these two areas in avian by locally disturbing neurogenesis originates a change in vascular patterning, suggesting that this particular growth path is controlled by the surrounding neural cells (Takahashi et al., 2015). Further, gain-of-function studies in chick embryos demonstrated that the stereotypical pattern along the neural progenitor area is achieved by the secretion of VEGF from the neural progenitors, attracting growing sprouts, but simultaneously endothelial cell response to VEGF is fine-tuned by sFLT1 secretion by ECs (Takahashi et al., 2015).

Notably, the degree of angiogenesis needs to be controlled as negative effects on the neural compartment can arise when too much VEGF is available. As shown in Himmels et al. (2017), MNs secrete VEGF and sFLT1 simultaneously to ensure that a correct level and timing of angiogenesis takes place. When this balance of factors is disrupted and excessive angiogenesis occurs, MNs are distributed incorrectly and MN axons leave the SC defasciculated (Himmels et al., 2017). When extrapolating this to situations, where axon regrowth is needed, it is possible that simply inducing regeneration of axons and angiogenesis is not enough to promote proper axon regrowth, but that angiogenesis needs to be limited to not disrupt the other process.

With respect to the specific pattern of arteries, veins, and capillaries, it is well-defined that in the adult SC the main arterial supply is achieved by the presence of segmental spinal arteries arising from the vertebral arteries, dorsal intercostal arteries, and lumbar arteries. Blood flows in the SC through these arteries and is further drained into the large and dorsal and posterior spinal veins in the SC (Farrar et al., 2015; Mazensky et al., 2017). Yet, it remains to be further characterized how the pattern of these three different vessel types appears during development.

## Common Molecular Factors in Development and Pathology

CNS pathologies are multifaceted and complex pathologies characterized by cell death, axon damage/degeneration, loss of vascular integrity, disruption of the BBB and blood-spinal cord barrier (BSCB), inflammation, and ECM (extracellular matrix) remodeling (Griffin and Bradke, 2020). The BBB and BSCB are sophisticated barrier systems in which ECs and their tight junctions play a central part in association with astrocyte end-feet, perivascular macrophages, pericytes, and basement lamina (Bartanusz et al., 2011). However, they are heterogenous in concern to their expression of barrier-specific proteins and their functional permeability. Compared to brain, ECs in the SC seem to have decreased expression of adherens junction (AJ) and tight junction (TJs) proteins and show a corresponding increase in permeability to low-molecular-weight tracers (Bartanusz et al., 2011).

It is by now understood that neuronal regeneration events during pathology and embryonic development by NPCs elicit very similar transcriptomic response making them fairly similar processes (Poplawski et al., 2020). Comparably, pathways that regulate vascular development and BBB properties are also active in pathological conditions. We provide here some examples of these molecules and their signaling pathways that play a significant role during vascularization and BBB formation in the SC and also during its pathology. Due to space limitations, we took as examples the experimental autoimmune encephalomyelitis (EAE), amyotrophic later sclerosis (ALS), and arteriovenous malformations (AVMs) in the SC.

Among the available experimental models to understand the BSCB pathology, the **EAE mouse model**, characterized by autoimmune attack to oligodendrocytes in the CNS leading to their loss and demyelination of axons, is one of the most commonly used (Tonra et al., 2001; Muller et al., 2005; Schellenberg et al., 2007; Aube et al., 2014). Multiple reports describe changes in endothelial proliferation, vessel morphology, and increased blood vessel density in the SC and brain with EAE (Kirk and Karlik, 2003; Roscoe et al., 2009; Seabrook et al., 2010). In addition, a recent study applying the EAE model in Claudin5-GFP reporter line (with ECs expressing GFP) shows that remodeling of TJs in ECs and paracellular BSCB leakage precedes the EAE disease onset. (Lutz et al., 2017). Changes in VEGF expression and increased levels of VEGF have been described in EAE and in multiple sclerosis (MS) patients (Proescholdt et al., 2002; Su et al., 2006; Shimizu et al., 2012) and may eventually be responsible for the increase in EC proliferation and vessel density as well as for the leaky barrier (Argaw et al., 2009; Luissint et al., 2012). Once the immune system gets activated, it further exacerbates VEGF signaling cascade ending into a feedback loop that would further promote BSCB leakage and inflammation (Argaw et al., 2006, 2012). Based on this increase in VEGF expression, one of the strategies to balance the VEGF availability, learned from developmental studies, would be to promote the expression of sFLT1 that could titrate the excess of VEGF from the system (Himmels et al., 2017; Wild et al., 2017).

As mentioned above, WNT ligands are known for their specific role in CNS angiogenesis and BBB formation (Stenman et al., 2008; Daneman et al., 2009). Consistent with its role as a co-receptor for WNT ligands, GPR124 also participates in BBB formation (Kuhnert et al., 2010; Anderson et al., 2011; Cullen et al., 2011). The WNT/β-catenin signaling pathway is important for adult BBB maintenance as shown in multiple reports (Tran et al., 2016; Chang et al., 2017; Griveau et al., 2018) and is activated in CNS endothelium also in EAE and human MS during the course of disease progression (Lengfeld et al., 2017; Niu et al., 2019). The re-activation of this pathway in pathological conditions may suggest a rapid endothelial response toward restoring the barrier properties of the damaged vessels. Consistent with this hypothesis, *in vivo* inhibition of WNT signaling in ECs exacerbated EAE pathology with increased mortality, greater infiltration of CD4+ T cells into the CNS and more drastic myelin loss (Lengfeld et al., 2017). Interestingly, in postnatal or adult mice conditional deletion of endothelial GPR124 resulted in no defects in CNS angiogenesis, BBB development or maintenance; making GPR124 dispensable for vascular homeostasis in adult CNS (Chang et al., 2017). However, deficiency of GPR124 in a pathological mouse model of ischemic stroke or glioblastoma leads to extensive BBB leakage and hemorrhage, microvascular damage accompanied by pericyte, ECM, and TJ deficits. Thus, similar as the re-activation of the WNT signaling pathway, the GPR124-WNT signaling axis is important in maintaining vascular homeostasis during injury in adult. Considering that BSCB leakage is a primary feature of several diseases, one approach to target leakage, suggested by those studies, and by the role of the WNT/β-catenin signaling in BBB formation during development, could be to promote the activation of this pathway in ECs in pathological conditions (Liu et al., 2014; Jia et al., 2019). This was already shown in the above mentioned GPR124 deletion mouse pathology-model, where EC-specific constitutive activation of WNT signaling *via* activated β-catenin restored the vascular defects (Chang et al., 2017). In line with this idea, it was recently also shown that the activation of β-catenin in ECs from circumventricular organs of the CNS, which under physiological conditions lack BBB properties and are permeable, results in a tightened BBB in those regions and augmented neuronal activity (Benz et al., 2019).

**Amyotrophic lateral sclerosis (ALS)** is another SC and brain related disease, where the progression of MN degeneration leads to muscle atrophy, paralysis, and death. In ALS, BSCB impairment is also shown (Taylor et al., 2016). The disruption of endothelial TJ proteins like ZO1, occludin, and claudin5 seems to be the primary cause of microhemorrhages, reduced microcirculation, prior to the MN degeneration and the inflammatory response (Zhong et al., 2008). There is increasing evidence suggesting that MN degeneration is not only due to intrinsic defects, but also that the surrounding cell types like microglia, astrocytes, oligodendrocytes, and ECs may also be involved. A variety of growth and neurotrophic factors are also reported to mediate the ALS pathology (Bruijn et al., 2004). Of note, VEGF and WNTs are well studied. Mice with reduced VEGF levels (*Vegf^δ/δ^* mice) present reduced neural vascular perfusion and progressive MN degeneration, mimicking the human ALS (Oosthuyse et al., 2001). Multiple studies have shown the importance of VEGF in decelerating the disease outcome and providing a protective effect for MNs by both maintaining proper vessel perfusion and by acting directly on MNs as a survival factor (Lange et al., 2016). The underlying pathophysiological mechanisms leading to the MN degeneration and fatal outcome observed in ALS also seems to be linked to WNTs (Chen et al., 2012; Yu et al., 2013; Tury et al., 2014; Gonzalez-Fernandez et al., 2016, 2020; Bhinge et al., 2017). SOD1^G93A^ ALS mice, as well as ALS patients, present a dysregulation in WNT signaling with upregulation or downregulation of certain ligands, receptors, and coreceptors depending on the distinct cell of the CNS analyzed (Gonzalez-Fernandez et al., 2020). As WNT ligands can act and exert different functions in different cellular context, close examination of their effect in astrocytes, neurons, and blood vessels in ALS conditions is required to understand their overall outcome. In this regard, upregulation of canonical WNT signaling seems to promote glial proliferation, that is, eventually neuroprotective during ALS (Chen et al., 2012; Li et al., 2013; Yu et al., 2013). In a similar manner, extrapolating from their role in blood vessel and BBB formation in the CNS, upregulated WNT ligands could be a defense mechanism to also promote a tighter BBB. However, their role in conjunction with the CNS vessels in this pathology needs further investigation.

**Arteriovenous malformations (AVMs)** are characterized by abnormal tangles of blood vessels connecting arteries and veins, where blood flow is thus shunted from arteries to veins without passing through a capillary network (Mouchtouris et al., 2015). While AVMs can develop anywhere in our body, they occur most often in the CNS (being more common in the brain than in the SC). Spinal AVMs are mainly represented during the adulthood, however, they may also appear as a juvenile form, which can be intramedullary, extramedullary, and extraspinal (Ferch et al., 2001). Most of the molecular studies on CNS AVMs have been in the brain and indicate that similar mechanisms involved in CNS vascularization are de-regulated in AVMs. Although not described in the SC AVMs, here, we will describe those main findings.

Multiple studies suggest active angiogenesis as a feature of AVMs. Similarly, inflammation is a major contributor toward the pathogenesis and active angiogenesis of AVMs. The inflammatory response triggered by hemodynamic factors and/or genetic predisposition in the formation and rupture of AVMs involves different cytokines (IL-6, IL-1β, TNFα, and IL-8), neutrophils, and macrophages (Mouchtouris et al., 2015) that eventually upregulate the expression of VEGF. IL-6, IL-1β, and TNFα also induce NF-kB expression, which further exacerbates VEGF levels (Angelo and Kurzrock, 2007). Moreover, it is possible that this AVM niche itself may contribute to the stimulation of pathological angiogenesis, where the occurrence of focal ischemia leads to a hypoxic environment that in turn leads to angiogenesis by the expression of VEGF and VEGFR1 (Rothbart et al., 1996; Koizumi et al., 2002; Jabbour et al., 2009; Mouchtouris et al., 2015; Gamboa et al., 2017). Worth to mention, the NF-kB-VEGF cascade in AVMs is also stimulated under hypoxia by HIF-1 (Ng et al., 2005). Consistently, VEGF has been reported in patients suffering from recurrent cerebral AVMs (Rothbart et al., 1996) and VEGF receptors (VEGFR1 and VEGFR2), matrix metalloproteinases-MMP9 and ECM proteins like Collagen IV (Rothbart et al., 1996; Pyo et al., 2000; Hashimoto et al., 2003; Gamboa et al., 2017) are further thought to play a primary role in the pathogenesis of AVMs. As Notch signaling plays a critical role in the determining the arterio-venous cell fate during vascular development, it is thus crucial in AVMs also (Murphy et al., 2008; ZhuGe et al., 2009; Tetzlaff and Fischer, 2018). Manipulations in the notch signaling pathway leads to development of hallmark features of AVMs in the brain (Murphy et al., 2008; Nielsen et al., 2014). Recently a few studies have also shed light on the role of Wnt signaling in AVMs. β-catenin gain-of-function in ECs can cause arterial defects, including the loss of venous marker expression, arterialization of veins, and formation of AVMs (Corada et al., 2010). These ECs show a strong increase in Dll4/Notch signaling along with reduced sprouting activity, indicating a requirement for fine-tuning Wnt and Notch signaling pathways in the pathogenesis of AVMs. Additionally, a very recent study on whole-exome RNA sequencing in human samples of brain AVMs also showed an activation of canonical Wnt signaling *via* the activity of increased FZD10 and MYOC (Huo et al., 2019). All in all, the current understanding in AVM pathology, diagnoses, and treatment is increasing, but the molecular players and its regulation require further investigation. In addition, whether the same molecular mechanisms that lead to AVMs in the brain are also active in spinal AVMs is also something that remains to be investigated.

These examples show that VEGF and WNT signaling, that are important for blood vessel growth and BBB formation, during development are also involved in distinct pathologies.

## Concluding Remarks

Research in the past recent years has provided further mechanistic insight into neuro-vascular interactions and into the importance of those interactions for proper development and function of the CNS. Here, studies in the SC have been fundamental. To advance in our understanding of SC development and function further studies focused on delineating angiogenic cues and potential bidirectional signaling pathways are needed. Spinal cord injuries are debilitating and fatal in many cases. Understanding SC development from a neural-vascular interaction perspective, and the potential reactivation or inhibition of those molecular signaling pathways in pathological conditions will be important for further developing therapeutic strategies.

## Author Contributions

JRV, BS, and CRA wrote the manuscript. All authors contributed to the article and approved the submitted version.

### Conflict of Interest

The authors declare that the research was conducted in the absence of any commercial or financial relationships that could be construed as a potential conflict of interest.
